# Effects of a clinically led combined with “sandwich” teaching on professional performance, learning engagement, and core competencies among cardiac surgery ICU nurses

**DOI:** 10.3389/fmed.2026.1864308

**Published:** 2026-08-20

**Authors:** Liqin Mo, Yunyun Zeng, Yunxiao Liang, Changfeng Huang

**Affiliations:** The First Affiliated Hospital of Guangxi Medical University, Nanning, China

**Keywords:** cardiac surgery, core competencies, intensive care unit, nurse education, sandwich teaching

## Abstract

**Objective:**

To investigate the effects of a clinically led framework combined “sandwich” teaching on theoretical knowledge, clinical skills, learning engagement, and core competencies of Cardiac Surgery Intensive Care Unit (CSICU) nurses.

**Methods:**

This study was a single center quasi experimental study, using the sequential cohort allocation method. From November 2024 to April 2025, a total of 60 eligible nurses from the CSICU were included. Nurses who completed training from November 2024 to January 2025 received traditional teaching, whereas those who completed training from February to April 2025 received the combined teaching intervention. Both groups completed the same 3 month, 18 lesson course. Before and after the training, the theoretical knowledge, clinical skills, learning engagement, and core competencies of the two groups of nurses were evaluated and compared.

**Results:**

In the intervention group, theoretical knowledge, critical care nursing skills, and specialized technical skills all improved significantly after training (*p* < 0.05). In the control group, only critical care nursing skills improved significantly; The changes in theoretical knowledge and specialized technical skills were not statistically significant. Post training scores for all three knowledge and skill outcomes were higher in the intervention group than in the control group (*p* < 0.05). Total learning engagement increased within the intervention group (*p* < 0.05), but the post training between group difference did not reach statistical significance (*p* = 0.066). No baseline differences were observed in the five core competency dimensions, whereas all five post training dimension scores were higher in the intervention group (*p* < 0.05).

**Conclusion:**

The clinically led combined “sandwich” teaching model was associated with greater improvements in theoretical knowledge, clinical skill performance, and core competencies among CSICU nurses, its effect on overall learning engagement remains uncertain. This teaching model may serve as an effective educational strategy for CSICU nursing training in specialized surgical settings.

## 1 Introduction

With the continuously increasing incidence of cardiovascular diseases in China, higher professional competency requirements have been placed on nursing staff working in the Cardiac Surgery Intensive Care Unit (CSICU) (1, 2). Critically ill patients after cardiac surgery often present with complex conditions and rapid clinical changes, involving multidisciplinary knowledge such as circulatory support, respiratory management, hemodynamic monitoring, post–cardiopulmonary bypass care, and multi-organ function assessment (3). In this context, the level of nurses’ professional competence—encompassing theoretical knowledge, clinical skills, and core competencies—directly determines the quality and safety of patient care. Nurses with strong competence are better equipped to recognize complications early, make rapid and accurate clinical decisions, and ensure continuity of multidisciplinary care, all of which are critical to improving patient outcomes in the CSICU. Therefore, CSICU nurses are required not only to possess a solid theoretical foundation but also to demonstrate rapid clinical judgment, emergency response capabilities, and effective teamwork skills (4, 5).

Conventional training dominated by classroom lectures can efficiently deliver standardized information, but it may provide limited opportunities to connect theory with evolving clinical situations, articulate clinical reasoning, or obtain iterative peer and instructor feedback. These limitations are particularly relevant in the CSICU, where nurses must transfer knowledge to complex procedures and time sensitive decisions rather than rely on factual recall alone. In recent years, various innovative teaching models have been introduced in nursing education. The clinically led teaching model, also known as the Observation–Teaching–Discussion (OTD) model, emphasizes clinical observation, theoretical instruction, and case based discussion (6, 7). This approach strengthens teaching that is driven by real clinical problems, thereby enhancing relevance and clinical urgency. Meanwhile, the “sandwich” teaching model integrates teacher guidance, interactive discussion, collaborative summarization, and theme deepening in a cyclic and progressive manner, allowing repeated alternation between theory and practice (8, 9). This model provides a novel educational strategy for professional talent development.

The integration of the clinically led model with the “sandwich” teaching approach may help create a more realistic, practice-oriented teaching environment. Therefore, this study aimed to apply a clinically led combined “sandwich” teaching model in the training of CSICU nurses and to evaluate its effects on nurses’ theoretical knowledge, clinical skills, learning engagement, and core competencies, thereby providing evidence for optimizing training programs in cardiac surgery intensive care nursing.

## 2 Subjects and methods

### 2.1 Participants

This was a single center, quasi experimental study with pretraining and post training assessments and sequential group allocation. The study was conducted in the CSICU of our hospital between November 2024 and April 2025. To reduce contamination between teaching approaches, eligible nurses enrolled during the first period (November 2024 to January 2025) formed the control group, whereas those enrolled during the second period (February to April 2025) formed the intervention group.

Inclusion criteria were as follows: (1) holding a valid nurse practice license; (2) voluntary participation in the study. Exclusion criteria included: (1) nurses not officially registered at our hospital, such as visiting or trainee nurses; (2) nurses temporarily assigned to the CSICU from other departments. Withdrawal criteria included: (1) failure to attend more than two training sessions for any reason.

The sample size was calculated *a priori* using PASS 25.0.3 (https://www.ncss.com/software/pass/). The total core competency score was selected as the primary outcome. Based on previous similar study, an expected standardized effect size of Cohen’s d = 0.75 was assumed. With a two-sided significance level of 0.05, a statistical power of 80%, and a 1:1 allocation ratio, the minimum required sample size was 29 participants per group. Allowing for an anticipated attrition or incomplete-assessment rate of approximately 10%, 32 participants were planned for recruitment in each group, resulting in a total target sample of 64 nurses, of whom 60 completed the study and were included in the final analysis.

### 2.2 Methods

#### 2.2.1 Training program

Both groups completed 18 training sessions over three months, with each session lasting 100 minutes, resulting in a total formal contact time of 30 hours. The same teaching team, curriculum domains, training duration, and assessment schedule were used for both groups. The teaching team was selected from the hospital’s critical care nursing faculty pool. All instructors held formal teaching qualifications, had a bachelor’s degree or higher, held a professional title of senior nurse, had more than 10 years of specialized clinical experience, and had completed relevant external training.

The teaching team developed the *CSICU Specialist Nurse Training Manual* according to the training objectives, job requirements, previous teaching experience, and the characteristics of specialist nurses. The manual was reviewed and revised by the head nurse and critical care nursing experts before implementation. It covered specialized theoretical knowledge and clinical practice training. The curriculum is summarized in Table 1.

**Table 1:** Training course content.

Category	Main modules	Specific content
Professional Theory	Basic management and security	1. Nursing organizational structure, personnel responsibilities, and core systems 2. Top 10 patient safety goals, critical care safety and quality goals 3. Nursing emergency plan, adverse event reporting process, and patient transportation safety 4. Humanistic care, nurse patient communication skills, and nursing document writing standards 5. Key points for risk assessment of critical patient nursing
	Theory of infection prevention and control	1. General Introduction to ICU Infection Prevention and Control 2. Prevention and Control Principles of Central Venous Catheter, Ventilator, and Catheter-related Infections (CLABSI/VAP/CAUTI) 3 Key points of nosocomial infection prevention and control in CRRT and ECMO treatment
	Critical illness knowledge	1. Nursing principles for severe cardiovascular diseases (acute heart failure, shock, arrhythmia, etc.) 2. Diagnosis and nursing of severe respiratory diseases (ARDS, pneumothorax, pulmonary embolism, etc.) 3. Nursing theories for congenital heart disease/acquired heart disease/large vessel surgery 4. Nursing points for other severe systemic diseases (acute upper gastrointestinal bleeding, cerebrovascular accidents, etc.)
	Interpretation of guidelines and standards	1. Expert consensus on delirium, sedation and analgesia, and enteral nutrition. 2. Clinical guidelines for ECMO, blood purification, aortic dissection and other techniques. 3. Group standards of the Chinese Nursing Association (such as artificial airway, prevention of pressure injuries, etc.)
Clinical practice	Basic operational skills	1. Basic life support (cardiopulmonary resuscitation, electric cardioversion) 2. Artificial airway care (tracheal intubation/incision coordination, oral care, airbag management) 3. Standardized specimen collection, use of multifunctional monitor and alarm processing 4. Medical temperature controller, warm air blower, nitric oxide inhalation therapy operation
	Specialized guardianship technology	1. Circulation monitoring (invasive arterial pressure, central venous pressure, pulmonary artery pressure monitoring) 2. Respiratory support (mechanical ventilation, non-invasive positive pressure ventilation, prone position ventilation, nasal high flow oxygen therapy) 3. Application and observation of vasoactive drugs, interpretation of arterial blood gas analysis 4. Intracranial pressure, intra-abdominal pressure monitoring, and temporary pacemaker application
	Advanced life support	1. ECMO nursing (V-V/V-A mode operation, complication management, emergency management) 2. IABP operation standards, maintenance and evacuation points 3. Perioperative management of ventricular assist devices 4. Blood purification technology (CRRT on/off, anticoagulation management, alarm handling); Peritoneal dialysis
	Specialized nursing operations	1. Combination of bronchoscopy treatment 2. Postoperative nursing of cardiac surgery (pipeline nursing, nutritional support, nasointestinal tube retention, volume management) 3. Prevention and management of physical restraint, pressure injury, and incontinence dermatitis in critically ill patients 4. Management of analgesia, sedation, and early activity implementation in critically ill patients

#### 2.2.2 Training interventions

The control group received a traditional training model that combined offline multimedia classroom lectures with clinical skills practice. Instructors delivered theoretical content and guided nurses through practical skill exercises according to the established training plan.

The intervention group received the clinically led combined “sandwich” teaching model. The clinically led component followed the OTD sequence. Nurses first observed real clinical cases and identified key clinical problems. Instructors then used these problems to organize theoretical teaching and connect new knowledge with clinical practice. During the discussion stage, the sandwich method structured small group discussion, cross group exchange, reporting, and instructor feedback. Thus, the sandwich process was integrated into the OTD sequence rather than delivered as a separate intervention, helping participants apply, reflect on, and consolidate what they had learned.

##### 2.2.2.1 Clinical observation stage

This stage represented the Observation component of the OTD framework. Three to five days before each theoretical session, instructors announced the upcoming topic and provided guiding questions concerning etiology, pathophysiology, clinical manifestations, treatment strategies, monitoring, and key nursing considerations. Nurses were encouraged to observe relevant cases during routine work and, when appropriate, communicate with physicians, charge nurses, and patients to refine clinically relevant information. Questions generated during this stage served as the starting point for the subsequent Teaching and Discussion phases.

##### 2.2.2.2 Theoretical instruction stage

This stage represented the Teaching component of the OTD framework. Instructors delivered the curriculum in a structured and hierarchical manner and explicitly linked theoretical content to clinical problems identified during observation. Nurses were encouraged to raise questions about the theoretical material and observed clinical issues. Guided questioning was used to promote analysis rather than passive reception of information. At the end of each session, the instructor presented a representative clinical case and formulated six key questions (A-F). Nurses reviewed relevant literature and prepared for the subsequent discussion. A brief preview of the next topic was also provided to support advance observation.

##### 2.2.2.3 Group discussion stage

This stage represented the Discussion component of the OTD framework and operationalized the cyclic sandwich process. It was conducted before the next theoretical session and used the six questions generated during the preceding Teaching phase. The standardized sequence was as follows:

1. Initial group discussion: Nurses in the intervention group were divided into six groups (Groups A–F), with five nurses in each group. Each group was assigned one specific question and was responsible for in depth discussion and analysis of that topic.
2. Cross group discussion: After completing the initial discussions, nurses were reorganized into new groups based on numerical labels (A1–E1, A2–E2, etc.). In the new groups, each nurse presented the findings of their original group, ensuring that all participants actively contributed and exchanged perspectives. The detailed methods of the two groupings are presented in Supplementary Tables S2, S3.
3. Group synthesis and reporting: Nurses then returned to their original groups to integrate feedback from cross group discussions. Each group summarized its assigned question and selected one representative to present the conclusions, with additional input from other group members as needed.
4. Instructor summary: Instructors reviewed and synthesized the discussion outcomes, providing clarification, supplementation, and targeted feedback on the six key questions.
5. “Fishbowl” discussion: Instructors introduced new case related questions to stimulate further discussion. One nurse from each group was selected to form a central discussion group, while the remaining participants observed, reflected, and contributed when appropriate. This step was designed to assess nurses’ learning outcomes and practical application abilities.
6. Final feedback and transfer: The instructor summarized key objectives, addressed difficult points and unresolved questions, and linked the discussion outcomes to subsequent clinical observation and practice.

**Figure 1:**
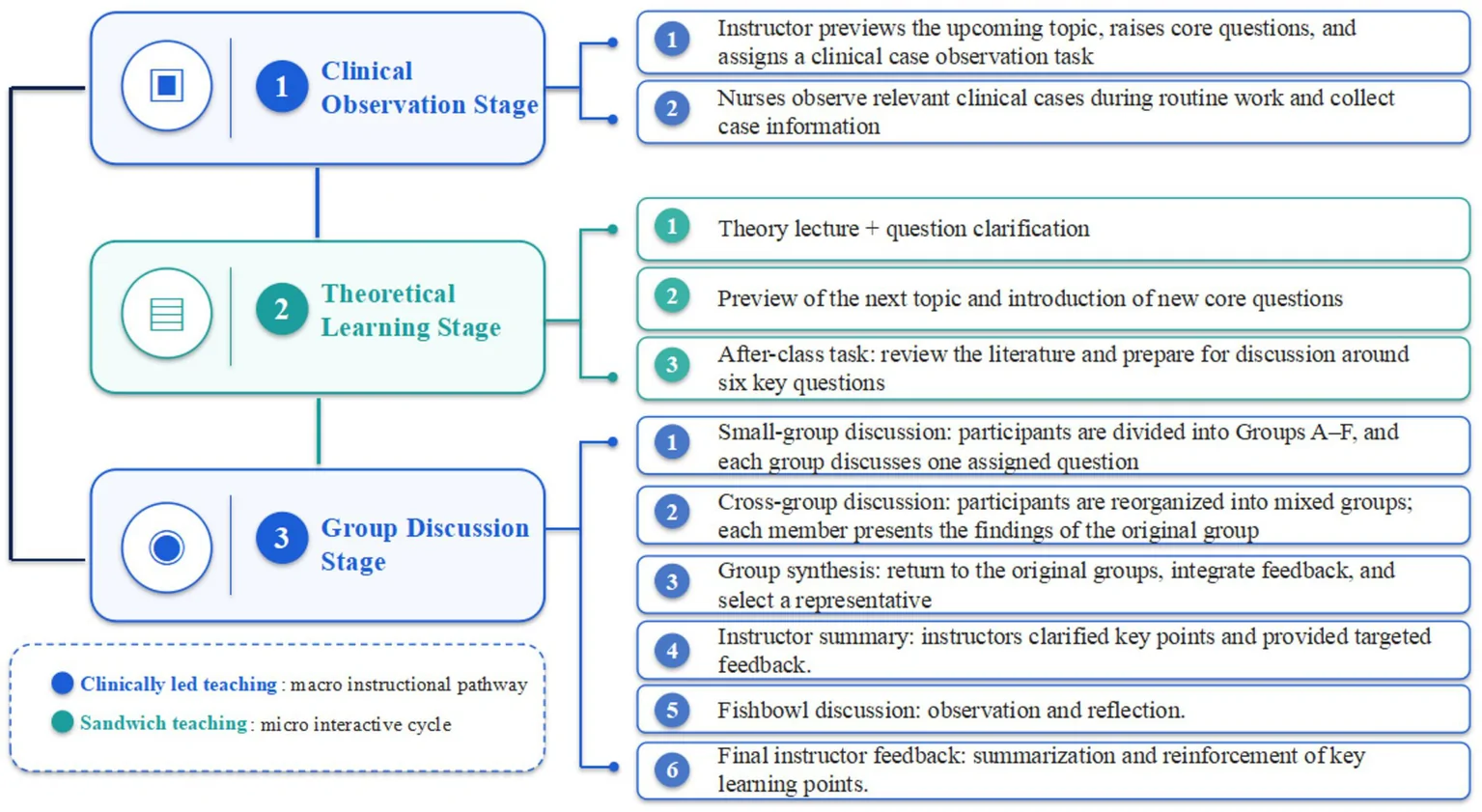
Operational workflow of the clinically led combined “sandwich” teaching mode.

The complete operational workflow is shown in Figure 1. Practical skills training in the intervention group combined theoretical explanation, peer discussion, and structured hands on exercises.

##### 2.2.2.4 Intervention implementation and quality control

The training manual and predefined course schedule were used to promote consistency across sessions. Before implementation, the teaching team reviewed the training objectives, course content, discussion sequence, case selection principles, and assessment requirements. The same instructor qualification standards, core curriculum, three month duration, and 18 session schedule were maintained across the two enrollment periods. Attendance was recorded throughout the study. Nurses were required to complete at least 16 of the 18 sessions to be included in the final analysis.

### 2.3 Outcome measures

#### 2.3.1 Specialized theoretical knowledge and skills assessment

Specialized training outcomes comprised a theoretical knowledge examination, a critical care nursing skills assessment, and a specialized technical skills assessment. All outcomes were measured before and after the three months program.

1. Theoretical knowledge assessment:

An online theoretical examination was administered using the Questionnaire Star (https://www.wjx.cn/) platform. The examination question bank was developed and uploaded by the teaching team. The examination duration was 60 min, with a total score of 100 points. All questions were in multiple choice format.

1. Critical care nursing skills assessment:

Six critical care nursing procedures were included: cardiopulmonary resuscitation, arterial blood sampling, gastrointestinal decompression, ventilator connection and operation, closed suctioning, and suction assisted oral care. One procedure was randomly selected for assessment, with a maximum score of 100 points.

1. Specialized technical skills assessment:

Four specialized technical procedures were assessed: assistance with bronchofibroscopy, initiation and discontinuation of continuous renal replacement therapy (CRRT), initiation and discontinuation of intra-aortic balloon pump (IABP) support, and assistance with extracorporeal membrane oxygenation (ECMO) initiation and withdrawal. One procedure was randomly selected for evaluation, with a full score of 100 points.

All practical skills assessments were conducted by independent assessors who were not involved in participant allocation, teaching delivery, or post-training data analysis. Participants were identified using coded study numbers, and the assessment sheets contained no information regarding group allocation. Assessors were not informed of the participants’ training conditions, and participants were instructed not to disclose their group assignment during the assessment. The same assessment environment, equipment, time limits, and procedural requirements were applied to both groups. Procedure specific scoring checklists were aligned with the training manual and institutional operating standards. Although task specific steps differed among procedures, the common assessment domains included pre-procedure assessment and preparation; patient identification, infection prevention, and safety verification; accuracy and sequence of critical procedural steps; patient monitoring and recognition or management of complications; communication and teamwork; and post-procedure management and documentation… Higher scores indicated better performance.

#### 2.3.2 Learning engagement

Learning engagement was assessed using the 17 item Utrecht Work Engagement Scale Student version (UWES-S-17) (10). The scale includes vigor (6 items), dedication (5 items), and absorption (6 items). Each item is rated on a 7 point Likert scale ranging from 0 (strongly disagree) to 6 (strongly agree), yielding a total score ranging from 0 to 102. Higher scores indicate greater levels of learning engagement. In our study, the total Cronbach’s *α* of this scale is 0.939, and the Cronbach’s α for each dimension range from 0.845 to 0.915.

#### 2.3.3 Core competencies of ICU nurses

The ICU Nurse Core Competence Scale was used to evaluate nurses’ core competencies before and after training (11). The scale comprises 74 items across five dimensions: professional basic knowledge (17 items), clinical practice ability (19 items), nursing management ability (13 items), professional development capability (13 items), and clinical judgment thinking ability (12 items). Items are rated using a 5 point Likert scale from 1 (“no competence”) to 5 (“highly competent”), with total scores ranging from 74 to 370. Higher scores reflect stronger core competencies. In our study, the total Cronbach’s *α* of this scale is 0.893, and the Cronbach’s α for each dimension range from 0.792 to 0.873.

### 2.4 Statistical analysis

Statistical analyses were performed using SPSS version 28.0. Continuous variables were expressed as mean ± standard deviation (SD), while categorical variables were presented as frequencies and percentages. Paired *t* tests were used for within group comparisons, and rank sum tests were applied for between group comparisons. A two tailed *p* value < 0.05 was considered statistically significant.

## 3 Results

### 3.1 Baseline characteristics

In each group, two participants were excluded from the final analysis due to incomplete training attendance or failure to complete the post training assessments. All remaining participants completed a minimum of 16 training sessions, meeting the predefined attendance threshold for inclusion in the final analysis. There were no statistically significant differences in general demographic and professional characteristics between the two groups at baseline (*p* > 0.05), indicating comparability between groups. Detailed information is presented in Table 2.

**Table 2:** Comparison of general information between two groups of critical care nurses in cardiac surgery (*n* = 60).

Project	Control group (*n* = 30)	Intervention group (*n* = 30)	*t/ x^2^*	*P* value
Age (years)	26.07 ± 3.49	27.10 ± 3.46	1.152	0.254
Gender			0.341	0.559
Male	9(30.00%)	7(23.33%)		
Female	21(70.00%)	23(76.67%)		
Work experience (years)			0.495	0.781
<2	4(13.33%)	6(20.00%)		
2 ~ 5	18(60.00%)	17(56.67%)		
6 ~ 10	8(26.67%)	7(23.33%)		

### 3.2 Specialized theoretical knowledge and skills assessment

In the intervention group, theoretical knowledge, critical care nursing skills, and specialized technical skills all improved significantly after training (*p* < 0.001). In the control group, critical care nursing skills improved significantly (*p* < 0.001), whereas changes in theoretical knowledge and specialized technical skills did not reach statistical significance. No significant between group differences were observed before training. After training, the intervention group had higher theoretical knowledge, critical care nursing skills, and specialized technical skills scores than the control group (*p* < 0.001; Table 3).

**Table 3:** Specialized theoretical and operational assessment scores of two groups of nurses before and after training (*n* = 60; score, ^−^*x* ± *s*).

Project	Grouping	Before training	After training	t/Z	*P*
Theoretical knowledge assessment	Control group	89.30 ± 4.10	90.93 ± 2.96	1.768	0.082
	Intervention group	90.30 ± 4.15	94.63 ± 2.70	3.919	<0.001
	Inter comparison (before training)			0.938	0.352
	Inter comparison (after training)			5.062	<0.001
Critical care nursing skills assessment	Control group	92.77 ± 2.01	94.83 ± 1.70	4.294	<0.001
	Intervention group	93.13 ± 2.02	96.53 ± 1.85	6.809	<0.001
	Inter comparison (before training)			0.706	0.483
	Inter comparison (after training)			3.700	<0.001
Specialized technical skills assessment	Control group	88.57 ± 4.44	90.30 ± 2.95	1.781	0.080
	Intervention group	86.87 ± 4.54	93.47 ± 2.71	6.836	<0.001
	Inter comparison (before training)			1.467	0.148
	Inter comparison (after training)			4.328	<0.001

### 3.3 Learning engagement of ICU nurses

Before training, total learning engagement scores did not differ significantly between groups (*p* = 0.965). After training, the control group showed no significant within group change (*p* = 0.919), whereas the intervention group showed a significant increase (*p* < 0.05). However, the post training between group difference in the total score did not reach statistical significance (*p* = 0.066). Dimension-specific analyses showed significantly higher post-training vigor (*p* < 0.05) and absorption (*p* < 0.05) scores in the intervention group, whereas the between-group difference in dedication remained nonsignificant (*p* = 0.442; Table 4).

**Table 4:** Comparison of ICU nurse learning engagement scores before and after training between two groups of nurses (*n* = 60; score, ^−^*x ± s*).

Project	Grouping	Before training	After training	t/Z	P
Vigor score	Control group	29.47 ± 7.15	29.60 ± 6.82	0.750	0.459
	Intervention group	29.57 ± 8.08	32.90 ± 5.59	5.079	<0.001
	Inter comparison (before training)			0.051	0.960
	Inter comparison (after training)			2.051	0.045
Dedication score	Control group	24.70 ± 4.81	24.87 ± 4.80	0.895	0.378
	Intervention group	24.60 ± 3.37	25.77 ± 4.18	2.495	0.019
	Inter comparison (before training)			0.093	0.926
	Inter comparison (after training)			0.774	0.442
Absorption score	Control group	29.57 ± 7.43	29.73 ± 7.44	0.867	0.393
	Intervention group	29.37 ± 6.89	33.20 ± 5.23	7.913	<0.001
	Inter comparison (before training)			0.108	0.914
	Inter comparison (after training)			2.089	0.041
Total score	Control group	83.73 ± 18.05	84.20 ± 17.54	0.102	0.919
	Intervention group	83.53 ± 17.23	91.87 ± 13.93	2.060	0.044
	Inter comparison (before training)			0.044	0.965
	Inter comparison (after training)			1.875	0.066

### 3.4 Core competencies of ICU nurses

No significant between-group differences were observed in the five core competency dimensions or the total score before training. Within the intervention group, all five dimension scores and the total score increased significantly after training. After training, the intervention group had significantly higher scores than the control group across all five dimensions and for the total core competency score (*p* < 0.05; Table 5).

**Table 5:** Comparison of core competency scores of ICU nurses before and after training between two groups of nurses (*n* = 60; score, ^−^*x ± s*).

Project	Grouping	Before training	After training	t/Z	P
Professional basic knowledge	Control group	51.80 ± 14.10	54.33 ± 14.34	0.690	0.493
	Intervention group	50.97 ± 11.46	60.72 ± 11.63	3.419	0.001
	Inter comparison (before training)			0.251	0.802
	Inter comparison (after training)			2.030	0.047
Clinical practice ability	Control group	70.27 ± 11.36	72.03 ± 13.01	0.560	0.577
	Intervention group	68.30 ± 10.94	78.13 ± 7.68	4.120	<0.001
	Inter comparison (before training)			0.683	0.497
	Inter comparison (after training)			2.288	0.026
Nursing management ability	Control group	37.60 ± 9.24	42.40 ± 10.57	1.872	0.066
	Intervention group	38.80 ± 10.90	48.48 ± 6.84	4.215	<0.001
	Inter comparison (before training)			0.460	0.647
	Inter comparison (after training)			2.742	0.008
Professional development capability	Control group	41.63 ± 11.36	44.00 ± 11.80	0.791	0.432
	Intervention group	41.77 ± 10.22	50.24 ± 8.97	3.517	0.001
	Inter comparison (before training)			0.048	0.962
	Inter comparison (after training)			2.396	0.020
Clinical judgment thinking ability	Control group	36.57 ± 7.98	40.60 ± 10.13	1.712	0.092
	Intervention group	37.83 ± 8.93	47.38 ± 6.74	4.484	<0.001
	Inter comparison (before training)			0.579	0.565
	Inter comparison (after training)			2.896	0.006
Total score	Control group	237.87 ± 23.72	253.37 ± 28.13	2.307	0.025
	Intervention group	237.67 ± 27.71	285.00 ± 29.34	6.532	<0.001
	Inter comparison (before training)			0.030	0.976
	Inter comparison (after training)			4.371	<0.001

## 4 Discussion

China has one of the world’s highest burdens of cardiovascular mortality, with a sustained upward trend and an increasing number of elderly, complex, and high risk patients (12, 13). Critically ill patients following cardiac surgery are characterized by rapid disease progression, frequent involvement of multiple organ systems, and a strong dependence on timely clinical interventions. These characteristics place high demands on CSICU nurses, who must possess not only solid theoretical knowledge and proficient technical skills but also sound clinical judgment and timely decision making abilities (14). Therefore, the training of CSICU nurses requires a dual track approach that strengthens both theoretical depth and practical competence, making the development of comprehensive and practice oriented training models a key direction for the advancement of cardiac surgical critical care nursing.

### 4.1 Effects of the clinically led combined “sandwich” teaching model on specialized competence

The improvement in theoretical knowledge and skills performance may be related to the integration of authentic clinical exposure, structured teaching, and repeated interactive learning. The intervention group showed significant improvements in theoretical knowledge, critical care nursing skills, and specialized technical skills after training. In the control group, only critical care nursing skills improved significantly, whereas the changes in theoretical knowledge and specialized technical skills did not reach statistical significance. In addition, the intervention group achieved significantly higher post-training scores on all three measures than the control group, suggesting that the combined teaching model may offer additional educational benefits beyond conventional training.

First, the OTD model emphasizes a “clinical first, theory second” approach, in which learning objectives are introduced in advance (15). Nurses actively observe clinical cases in authentic clinical settings and return to the classroom with authentic clinical questions. Early exposure to clinical scenarios enhances the contextualization and relevance of learning, transforming abstract concepts such as pathophysiology and treatment strategies into concrete clinical experiences (16, 17). This process facilitates the construction of a stable and meaningful knowledge framework.

Second, the “sandwich” teaching model employs a progressive structure involving instructor led teaching, group discussion, cross group exchange, and collective reporting. This repeated engagement to key concepts across different cognitive levels—comprehension, articulation, and reorganization—promotes deeper learning (18). Multidimensional discussion not only reinforces factual knowledge but also facilitates information reconstruction, thereby strengthening logical reasoning and coherence of clinical reasoning (19).

Finally, the clinically led model significantly enhanced nurses’ practical skills. Traditional skills training can emphasize the sequence of an operation without requiring learners to explain why specific actions are appropriate for a given clinical condition. In the combined model, case observation and discussion were linked to hands on practice, which may have helped participants connect technical execution with clinical reasoning. Nevertheless, only one randomly selected procedure from each skill category was assessed at each evaluation; these scores may not represent competence across the full range of procedures.

### 4.2 Effects on learning engagement of CSICU nurses

This study suggests that the clinically led, integrated “sandwich” teaching model enhanced specific dimensions of learning engagement among nurses in the CSICU. Within the intervention group, total learning engagement scores increased significantly from baseline to post intervention (*p* < 0.05), whereas no statistically significant change was observed in the control group. However, the between group difference in post intervention total engagement scores did not reach statistical significance (*p* = 0.066). Further analysis showed that the model did not affect all dimensions of engagement equally. After training, the intervention group had significantly higher vigor and absorption scores than the control group, whereas dedication did not differ significantly.

Learning engagement is a multidimensional construct comprising vigor (characterized by energy, resilience, and persistence in learning), dedication (reflecting enthusiasm, perceived meaningfulness, and identification with learning), and absorption (defined as full concentration and immersion in learning activities) (20). The observed improvement in vigor may be attributable to the clinical anchoring of the OTD framework. In the combined mode, nurses first engaged with authentic clinical cases, identified context specific challenges, and subsequently received theory based instruction. This sequence shifted learning from passive knowledge acquisition toward active, problem driven inquiry. By explicitly linking instructional content to daily clinical responsibilities, the model likely strengthened perceptions of relevance and utility—factors known to enhance greater effort and motivation. Consistent with prior evidence, case based pedagogy has been shown to foster active participation, reflective self-assessment, clinical decision making, and deeper cognitive processing in nursing education.

The enhancement in absorption appears primarily driven by the iterative, multi phase “sandwich” discussion structure. Participants engaged in sequential, scaffolded activities—including small group deliberation, inter group synthesis, plenary reporting, fishbowl observation, and structured instructor feedback (21). Each phase demanded sustained cognitive engagement: listening critically, analyzing diverse perspectives, formulating responses, and integrating insights. Such recurrent intellectual involvement likely promoted sustained attentional focus and deeper experiential immersion. In contrast, conventional lecture based instruction affords limited opportunities for continuous interaction; prolonged passive listening is associated with declining attentional resources over time. Empirical support for active learning underscores that structured dialogue, collaborative problem solving, and peer mediated knowledge construction yield superior learning outcomes relative to didactic delivery. Prior studies further have also shown that sandwich style instruction enhances self-directed learning capacity, critical thinking proficiency, and overall course satisfaction—reinforcing the pedagogical value of repeated, interactive discourse (22, 23).

Although dedication scores improved significantly within the intervention group (*p* < 0.05), the between group difference remained nonsignificant. This pattern suggests that dedication—being closely tied to professional identity formation, long term value attribution, and affective commitment to learning—may be less responsive to brief, skill focused educational interventions than vigor or absorption (24). While the OTD and ‘sandwich’ may rapidly elevate task specific energy and situational focus, shifts in enduring professional attitudes and meaning making processes typically require extended exposure, reinforcement, and supportive organizational conditions. Moreover, both groups received identical curricular content, instructional duration, and clinical competency training—implying that the control condition itself may have conferred modest gains in learning awareness, thereby attenuating intergroup disparities in dedication.

The nonsignificant between group difference in total engagement scores likely reflects the composite nature of the construct: although vigor and absorption showed robust intervention effects, the absence of a statistically discernible impact on dedication diminished the overall magnitude of the between group effect. Furthermore, learning engagement in high acuity clinical settings is subject to multiple contextual moderators—including heavy patient workload, rotating shift schedules, cumulative fatigue, individual motivational dispositions, availability of protected learning time, and institutional facilitation of professional development (25). CSICU nurses routinely manage life threatening conditions, rendering them highly susceptible to interruptions during preparatory, reflective, and collaborative learning phases—potentially diluting the model’s efficacy. Additionally, the relatively small sample size and moderate to high variability in baseline and post intervention engagement scores may have constrained statistical power.

Collectively, results support the model’s potential to meaningfully augment key engagement indicators; however, definitive conclusions regarding its comparative effectiveness and durability over time warrant validation through larger scale, multicenter trials with extended follow up periods and robust process evaluation.

### 4.3 Effects on core competencies of CSICU nurses

Core competence reflects nurses’ ability to integrate professional knowledge, technical skills, clinical judgment, decision making, and teamwork in practice. It is closely related to nursing quality and patient safety (26, 27). Higher core competence may help nurses respond more effectively to rapidly changing clinical conditions, identify risks earlier, and make timely and appropriate decisions in emergency situations (28).

In this study, the intervention group achieved higher post training overall core competence scores and higher scores across all five dimensions, including professional basic knowledge, clinical practice, nursing management, professional development, and clinical judgment, compared with the control group (*p* < 0.05). These results suggest that the clinically led combined “sandwich” teaching model may promote broader competency development rather than improvement in knowledge alone. Traditional one way teaching mainly emphasizes information delivery and provides limited opportunities for nurses to apply knowledge, explain decisions, or reflect on clinical problems. In contrast, the present model begins with real clinical cases and requires nurses to identify problems before theoretical instruction. This sequence may strengthen the connection between knowledge and practice and help nurses understand why specific assessments, interventions, and nursing priorities are required.

The improvement in professional basic knowledge may be related to the structured teaching that followed clinical observation. Nurses entered the teaching session with specific questions generated from real cases, which may have helped them organize fragmented knowledge and understand its clinical relevance. The increase in clinical practice competence may reflect repeated links between patient information, nursing procedures, monitoring findings, and bedside interventions. Instructors’ explanations and feedback also provided opportunities to correct misunderstandings and clarify procedural standards.

Clinical judgment may have benefited from repeated analysis of patient condition changes, treatment responses, monitoring trends, and potential complications. Exposure to complex and dynamic cases encouraged nurses to compare alternative explanations, identify risks, and justify their decisions, thereby supporting clinical reasoning and anticipatory thinking (29, 30). Nursing management competence may have been strengthened through small group discussion, cross group reporting, task allocation, and collective decision making. These activities required participants to communicate clearly, coordinate with others, prioritize problems, and integrate different viewpoints, which are essential abilities in the CSICU environment.

The improvement in professional development may be associated with reflection, peer learning, and active participation. During multiple discussion rounds, nurses were required to express their views, respond to questions, and reconsider their own reasoning after receiving feedback. This process may have increased awareness of individual knowledge gaps and encouraged more active learning and self-improvement. Cross group exchange also exposed participants to different problem solving approaches and may have broadened their understanding of clinical practice (31).

Overall, the model combined clinical triggers, structured instruction, repeated discussion, and reflection. Through this process, nurses moved from passive recipients of information to active participants, problem solvers, and reflective practitioners. This may explain why improvements were observed across several dimensions of core competence.

## 5 Limitations

This study has several limitations. Most outcomes were quantitative, and both the theoretical examination and procedural checklists were developed locally without external validation. In addition, only one randomly selected procedure from each skill category was assessed, so the results may not capture nurses’ overall practical competence. Learning engagement and core competence were measured using self-reported scales, while more objective outcomes—such as workplace performance, long-term knowledge retention, nursing quality, and patient outcomes—were not examined.

Second, no qualitative or formal process-evaluation data were collected. We therefore could not fully assess participants’ learning experiences, instructor feedback, intervention fidelity, or implementation barriers. The multi-stage discussion process may also increase session length, preparation requirements, and facilitator workload. Prolonged training may contribute to fatigue, reduced attention, scheduling conflicts, or lower participation, particularly among nurses with heavy clinical duties. However, these potential negative effects were not directly measured in this study.

Third, the study was conducted in a single CSICU at a tertiary hospital, which limits generalizability. Staffing, workload, protected teaching time, instructor qualifications, clinical case diversity, surgical volume, and access to ECMO, IABP, and CRRT may differ across hospitals and regions. These factors may influence both implementation and educational outcomes. The effect may also vary by clinical experience. Novice nurses may require more structured guidance, whereas experienced nurses may benefit more from complex case discussion but show smaller score changes because of higher baseline competence. The sample size did not allow reliable subgroup analyses by years of experience or professional seniority.

Future studies should therefore recruit nurses from hospitals of different levels and include participants with a broader range of clinical experience. It would also be useful to compare the full course with a shorter version to see whether similar outcomes can be achieved with less time and fewer resources. For example, the initial and cross-group discussions could be merged, while the separate reporting and fishbowl stages could be replaced by a single structured plenary session. A mixed offline–online format may further ease the time burden. Process evaluation, qualitative interviews, and longer follow-up are also needed to examine feasibility, participant burden, knowledge retention, workplace performance, nursing quality, and implementation costs.

## 6 Conclusion

In this study, the clinically led combined “sandwich” teaching model was associated with better post training performance across several educational outcomes among CSICU nurses. Compared with traditional teaching, the model was associated with higher theoretical knowledge, critical care nursing skill, specialized technical skill, and self-reported core competency scores. These findings suggest that integrating clinical observation, structured instruction, and repeated discussion may strengthen the connection between theoretical knowledge and clinical practice while supporting technical performance, clinical judgment, nursing management, and professional development.

The model also appeared to influence learning engagement, although the findings were less consistent than those for knowledge, skills, and competencies. Total engagement improved within the intervention group, but the post training difference between groups was not statistically significant. Dimension specific analyses suggested clearer differences in vigor and absorption than in dedication.

Overall, the model may provide a useful approach to the continuing education of CSICU nurses, particularly for integrating knowledge acquisition, practical training, clinical reasoning, and collaborative learning. However, the findings remain preliminary because they were derived from a small, single center study. Concurrent multicenter studies should confirm these educational effects and determine whether seniority stratified training, shortened sandwich cycles, and blended offline–online observation can reduce implementation burden while maintaining sustained educational and clinical benefits.
