# Supplementary material for: Effects of a clinically led combined with “sandwich” teaching on professional performance, learning engagement, and core competencies among cardiac surgery ICU nurses
Source: Front Med (Lausanne). 2026 Aug 20;13:1864308. doi: 10.3389/fmed.2026.1864308 (PMC13538001; doi:10.3389/fmed.2026.1864308)
Supplement: Supplementary file 1 [file Table_1.docx]

**Title:** Effects of a Clinically Led Combined with “Sandwich” Teaching on Professional Performance, Learning Engagement, and Core Competencies among Cardiac Surgery ICU Nurses

**List of Supporting Information**

**Table S1.** Comparison of General Information between Two Groups of Critical Care Nurses in Cardiac Surgery

**Table S2.** Original subgroups of panel discussion

**Table S3.** New subgroups for cross-study

Table S1. Comparison of General Information between Two Groups of Critical Care Nurses in Cardiac Surgery (initial, n=64)

| Project | Control group（n=32） | Observation group（n=32） | *t/ x^2^* | *P* value |
| --- | --- | --- | --- | --- |
| Age（years） | 26.09±3.38 | 27.00±3.37 | 1.074 | 0.287 |
| Gander |  |  | 0.721 | 0.396 |
| Male | 10（31.25%） | 7（21.88%） |  |  |
| Female | 22（68.75%） | 25（78.12%） |  |  |
| Work experience (years) |  |  | 0.505 | 0.777 |
| <2 | 4（12.50%） | 6（18.75%） |  |  |
| 2~5 | 20（62.50%） | 18（56.25%） |  |  |
| 6~10 | 8（25.00%） | 8（25.00%） |  |  |

Table S2. Original subgroups of panel discussion

|  | Group A | Group B | Group C | Group D | Group E | Group F |
| --- | --- | --- | --- | --- | --- | --- |
| 1 | A-1 | A-2 | A-3 | A-4 | A-5 | A-6 |
| 2 | B-1 | B-2 | B-3 | B-4 | B-5 | B-6 |
| 3 | C-1 | C-2 | C-3 | C-4 | C-5 | C-6 |
| 4 | D-1 | D-2 | D-3 | D-4 | D-5 | D-6 |
| 5 | E-1 | E-2 | E-3 | E-4 | E-5 | E-6 |
|  | Question-1 | Question-2 | Question-3 | Question-4 | Question-5 | Question-6 |

Table S3. New subgroups for cross-study

|  | 1 | 2 | 3 | 4 | 5 | 6 | Question |
| --- | --- | --- | --- | --- | --- | --- | --- |
| Group A | A-1 | A-2 | A-3 | A-4 | A-5 | A-6 | Original questions |
| Group B | B-1 | B-2 | B-3 | B-4 | B-5 | B-6 | Original questions |
| Group C | C-1 | C-2 | C-3 | C-4 | C-5 | C-6 | Original questions |
| Group D | D-1 | D-2 | D-3 | D-4 | D-5 | D-6 | Original questions |
| Group E | E-1 | E-2 | E-3 | E-4 | E-5 | E-6 | Original questions |

Note: For example: Group A: A1 reports on ‘Question-1’; A2 reports on ‘Question-2’; A3 reports on ‘Question-3’; A4 reports on ‘Question-4’; A5 reports on ‘Question-5’; A6 reports on ‘Question-6’.
